# Multi-agent Safety Verification Using Symmetry Transformations

**DOI:** 10.1007/978-3-030-45190-5_10

**Published:** 2020-03-13

**Authors:** Hussein Sibai, Navid Mokhlesi, Chuchu Fan, Sayan Mitra

**Affiliations:** 8 Johannes Kepler University, Linz, Austria; 9 University of Birmingham, Birmingham, UK; University of Illinois, Urbana, IL 61801 Champaign, USA

## Abstract

We show that symmetry transformations and caching can enable scalable, and possibly unbounded, verification of multi-agent systems. Symmetry transformations map any solution of the system to another solution. We show that this property can be used to transform cached reachsets to compute new reachsets, for hybrid and multi-agent models. We develop a notion of a *virtual system* which defines symmetry transformations for a broad class of agent models that visit waypoint sequences. Using this notion of a virtual system, we present a prototype tool *CacheReach* that builds a cache of reachsets, in a way that is agnostic of the representation of the reachsets and the reachability analysis method used. Our experimental evaluation of *CacheReach* shows up to 64% savings in safety verification computation time on multi-agent systems with 3-dimensional linear and 4-dimensional nonlinear fixed-wing aircraft models following sequences of waypoints. These savings and our theoretical results illustrate the potential benefits of using symmetry-based caching in the safety verification of multi-agent systems.

### 1 Introduction

As the cornerstone for safety verification of dynamical and hybrid systems, reachability analysis has attracted attention and has delivered automatic analysis of automotive, aerospace, and medical applications [2,24,17,11]. Notable advances from the last few years include the development of the generalized star data-structure [14] and the HyLaa tool [3] which can analyze massive linear models [4]; Taylor model based reachability analysis algorithms for nonlinear systems and their implementations in Flow* [7]; and a simulation-based algorithm that guarantees locally optimal precision [15].

Exact symbolic reachability analysis of nonlinear models is generally hard. One prominent approach is based on generalizing individual behaviors or simulations to cover a whole set of behaviors. The idea was pioneered in [10] and implemented in Breach [9] with sound generalization guarantees for linear models based on *sensitivity analysis*. Subsequently, the idea has been significantly extended to cover nonlinear, hybrid, and black-box models and it has been implemented in tools like C2E2 and DryVR [12,19,17,16].

In all of the above, a single behavior $\xi$ of the system from an initial state, is generalized to a *compact set* of *neighboring* behaviors that contains all the behaviors starting

* The authors are supported by a research grant from The Boeing Company and a research grant from NSF (CPS 1739966). We would like to thank John L. Olson and Arthur S. Younger from The Boeing Company for valuable technical discussions.

from a small neighborhood around the initial state of $\xi$. Thus, the computed neighboring set of behaviors always contains $\xi$ and its size is determined by the algorithms for sensitivity analysis. In contrast, the type of generalization we pursue here uses *symmetry transforms* on the state space. Given a group $\Gamma$ of operators on the state space, and a single behavior $\xi$, we can generalize $\xi$ to $\gamma(\xi)$, for each $\gamma \in \Gamma$. Symmetry transformations can be applied to sets of behaviors symbolically. Not only can this type of generalization work in conjunction with sensitivity analysis, it captures structural properties of the system that make behaviors similar in a way that is not covered by sensitivity analysis.

In our recent work [29], we showed how symmetry transforms can be used to produce new reachsets from other previously computed reachsets for *non-parameterized* dynamical systems. In this paper, we introduce the use of symmetry transforms of *parameterized* dynamical systems for safety verification. We present an algorithm `symComputeReachtube` (Algorithm 1) which caches and reuses reachsets, avoiding repeating expensive computations. We show how an infinite number of reachsets can be obtained by transforming a single one using symmetry transforms (Corollary 2). Building on it, we provide unbounded time safety guarantees using finite cached safety checking results (Theorem 6).

The key contributions of this paper are as follows.

First, we show how symmetry transformations for parameterized dynamical systems can be used to compute reachable states (Theorem 2). Going well beyond the previous theory [29], this enables *cached reachtubes* to be reused for verification across different modes and across multiple agents.

We develop a notion of *virtual system* (Section 4) which automatically defines symmetry transformations for a broad swathe of hybrid and dynamical systems modeling agents visiting a sequence of waypoints (see Theorem 3 and Examples 3 and 4). That is, reachability analysis of a multi-agent system, with possibly different dynamics and different parameters, can be performed in a common transformed coordinate system, and thus, increases the possibility of reuse. We show how this principle can make it possible to verify systems over unbounded time and with infinite number of agents (Theorem 6), provided that no new unproven scenarios appear for the virtual system.

We present a prototype implementation of a tool that uses `symComputeReachtube`. We name it CacheReach. It builds a cache of reachtubes for the virtual system, from different sets of initial states. In performing reachability analysis of a multi-agent hybrid or dynamical system, for each agent and each mode, the algorithm proceeds as follows: (1) transform the initial set $X$ to an initial set of the virtual system to get $\gamma(X)$. (2) If the transformed set $\gamma(X)$ has already been stored in the cache, then extract it and apply $\gamma^{-1}$ to get the actual reachset. (3) Otherwise, compute the reachset from $\gamma(X)$ and cache it. Our algorithm `symComputeReachtube` and its implementation in CacheReach are agnostic of the representation of the reachsets and the reachability analysis subroutine, and therefore, any of the ever-improving libraries can be plugged-in for step 3.

Our experimental evaluation of CacheReach shows safety verification computation time savings of up to 64% on scenarios with multiple agents with 3-dimensional linear and 4-dimensional nonlinear fixed-wing aircraft model following sequences of waypoints. These savings illustrate the potential benefits of using symmetry transformations and caching in the safety verification of multi-agent systems.

### 2 Model and problem statement

*Notations.* We denote by $\mathbb{N}$, $\mathbb{R}$, and $\mathbb{R}^{\geq 0}$ the sets of natural numbers, real numbers and non-negative reals. Given a finite set $S$, its cardinality is denoted by $|S|$. Given $N \in \mathbb{N}$, we denote by $[N]$ the set $\{1, \ldots, N\}$. Given a vector $v \in \mathbb{R}^n$ and a set $L \subseteq [n]$, we denote the projection of $v$ to the indices in $L$ by $v[L]$. We define an $n$-dimensional hyper-rectangle by a 2d-array specifying its bottom-left and upper-right corners. We denote the projection of a hyper-rectangle $H$ on the set of dimensions $L$ by $H[L]$. Given a function $\gamma : \mathbb{R}^k \rightarrow \mathbb{R}^k$ and a set $S \subseteq \mathbb{R}^k$, we abuse notation and define $\gamma(S) = \{\gamma(x) \mid x \in S\}$. Moreover, given $S \in 2^{\mathbb{R}^k} \times \mathbb{R}^{\geq 0}$, we define $\gamma(S) = \{(\gamma(X), t) \mid (X, t) \in S\}$.

#### 2.1 Agent mode dynamics

In this section, we define the syntax and semantics of the model that determines the dynamics of an agent. We present the syntax first.

**Definition 1 (syntax).** *The agent dynamics are defined by a tuple $A = \langle S, P, f \rangle$, where $S \subseteq \mathbb{R}^n$ is its state space, $P \subseteq \mathbb{R}^m$ is its parameter or mode space, and the dynamic function $f : S \times P \rightarrow S$ that is Lipschitz in the first argument.*

The semantics of an agent dynamics is defined by trajectories, which describe the evolution of states over time.

**Definition 2 (semantics).** *For a given agent $A = \langle S, P, f \rangle$, we call a function $\xi : S \times P \times \mathbb{R}^{\geq 0} \rightarrow S$ a* trajectory *if $\xi$ is differentiable in its third argument, and given an initial state $\mathbf{x}_0 \in S$ and a mode $p \in P$, $\xi(\mathbf{x}_0, p, 0) = \mathbf{x}_0$ and for all $t > 0$,*

$$\frac{d\xi}{dt}(\mathbf{x}_0, p, t) = f(\xi(\mathbf{x}_0, p, t), p). \tag{1}$$

*We say that $\xi(\mathbf{x}_0, p, t)$ is the state of $A$ at time $t$ when it starts from $\mathbf{x}_0$ in mode $p$.*

Given an initial state $\mathbf{x}_0 \in S$ and mode $p \in P$, the trajectory $\xi(\mathbf{x}_0, p, \cdot)$ is the unique solution of the ordinary differential equation (ODE) (1) since $f$ is Lipschitz continuous.

Given a compact initial set $K \subseteq S$, a parameter $p \in P$, the set of *reachable states* of $A$ over a time interval $[ftime, etime]$ is defined as

$$\texttt{Reach}(K, p, [ftime, etime]) = \{\mathbf{x} \in S \mid \exists \mathbf{x}_0 \in K, t \in [ftime, etime], \mathbf{x} = \xi(\mathbf{x}_0, p, t)\}. \tag{2}$$

We let $\texttt{Reach}(K, p, t)$ denote the set of reachable states at time $t$. Unbounded reachset from $K$ and $p$ is $\texttt{Reach}(K, p, [ftime, \infty))$.

The *bounded time safety verification* problem requires one to check if any state reachable by $A$ for a given initial set $K$ and mode $p$ is unsafe within a given time bound. That is, given a time bound $T > 0$, $p \in P$, and an unsafe set $U \subseteq S$, we want to check whether $\texttt{Reach}(K, p, [0, T]) \cap U = \emptyset$.

#### 2.2 Reachtubes

Computing reachsets exactly is theoretically hard [22]. There are many reachability analysis tools [8,1,3] that can compute bounded-time over-approximations of the reachsets. Generally, given an initial set $K$ for a set of ODEs, these tools can return a sequence of sets that contain the exact reachset over small time intervals. Motived by this, we define reachtubes as sequences of time-annotated over-approximations of exact reachsets:

**Definition 3.** *For a given agent $A = \langle S, P, f \rangle$, an initial set $K \subseteq T$, a mode $p \in P$, and a time interval $[ftime, etime]$, a $(K, p, [ftime, etime])$-reachtube* $\texttt{ReachTb}(K, p, [0, T])$ *is a sequence $\{(X_i, [\tau_{i-1}, \tau_i])\}_{i=1}^{j}$ such that* $\texttt{Reach}(K, p, [\tau_{i-1}, \tau_i]) \subseteq X_i$*, and $\tau_0 = ftime < \tau_1 < \cdots < \tau_j = etime$. Without loss of generality, we assume equal separation between the time points, i.e. $\exists\ \tau_s > 0, \forall i \in [j], \tau_i - \tau_{i-1} = \tau_s$.*

For a given $(K, p, [ftime, etime])$-reachtube *rtube*, we denote its parameters by *rtube.K*, *rtube.p*, *rtube.ftime*, and *rtube.etime*, respectively, and its cardinality by *rtube.len*.

We define union, truncation, concatenate, and time-shift operators on reachtubes. Fix $rtube_1 = \{(X_{i,1}, [\tau_{i-1,1}, \tau_{i,1}])\}_{i=1}^{j_1}$ and $rtube_2 = \{(X_{i,2}, [\tau_{i-1,2}, \tau_{i,2}])\}_{i=1}^{j_2}$ to be two reachtubes, where $j_1 = rtube_1.len$ and $j_2 = rtube_2.len$. If $\tau_{i,1} = \tau_{i,2}$ for all $i \in [\min(j_1, j_2)]$, we say they are *time-aligned*. Without loss of generality, assume that $j_1 \leq j_2$. The operators are defined as follows:

- *timeShift*$(rtube_1, t_s) = \{(X_{i,1}, [t_s + \tau_{i-1,1}, t_s + \tau_{i,1}])\}_{i=1}^{j_1}$,
- *union*: $rtube_1 \cup rtube_2 = \{(X_{i,1} \cup X_{i,2}, [\tau_{i-1,1}, \tau_{i,1}])\}_{i=1}^{j_1} \cup \{(X_{i,2}, [\tau_{i-1,2}, \tau_{i,2}])\}_{i=j_1+1}^{j_2}$,
- *concatenation*: $rtube_1 \frown rtube_2 = rtube_1 \cup \{(X_{i,2}, [\tau_{j_1,1} + \tau_{i-1,2}, \tau_{j_1,1} + \tau_{i,2}))\}_{i=1}^{j_2}$,
- *truncate*$(rtube_1, t_c) = \{(X_{i,1}, [\tau_{i-1,1}, \tau_{i,1}])\}_{i=1}^{k}$, where $\tau_{k,1} \geq t_c$ and $\tau_{k-1,1} < t_c$.

A *simulation* of system (1) is a reachtube with $X_0$ being a singleton state $x_0 \in K$. That is, a simulation is a representation of $\xi(x_0, p, \cdot)$. Several numerical solvers can compute such simulations as VNODE-LP[1] and CAPD Dyn-Sys library [2].

*Example 1 (Fixed-wing aircraft following a single waypoint).* Consider an agent with state space $S = \mathbb{R}^4$, parameter space $P = \mathbb{R}^4$, and $f : S \times P \to S$ defined as follows: for any $\mathbf{x} \in S$ and $p \in P$,

$$f(\mathbf{x}, p) = [\frac{T_c - c_{d1}\mathbf{x}[0]^2}{m}, \frac{g}{\mathbf{x}[0]} \sin\phi, \mathbf{x}[0]\cos\mathbf{x}[1], \mathbf{x}[0]\sin\mathbf{x}[1]],$$

where $T_c = k_1 m(v_c - \mathbf{x}[0])$, $\phi = k_2 \frac{v_c}{g}(\psi_c - \mathbf{x}[1])$, $\psi_c = \arctan_2(\frac{\mathbf{x}[2]-p[2]}{\mathbf{x}[3]-p[3]})$, and $k_1, k_2, m, g$, $c_{d1}$, and $v_c$ are positive constants. The agent models a fixed-wing aircraft starting from a waypoint and following another in the 2D plane: $\mathbf{x}[0]$ is its speed, $\mathbf{x}[1]$ is its heading angle, $(\mathbf{x}[2], \mathbf{x}[3])$ is its position in the plane, $[p[0], p[1]]$ is the position of the source waypoint, and $(p[2], p[3])$ is the position of the destination one. Note that the source waypoint does not affect the dynamics, but will be useful later in the paper.

[1] http://www.cas.mcmaster.ca/~nedialk/vnodelp/

[2] http://capd.sourceforge.net/capdDynSys/docs/html/odes_rigorous.html

## 3 Symmetry and Equivariant Dynamical Systems

Symmetry plays a fundamental role in the analysis of dynamical systems. It has been used for studying stability of feedback systems [25], designing observers [5] and controllers [30], and analyzing neural networks [20]. In this section, we present definitions of symmetries and their implications on systems that posses them.

### 3.1 Symmetry of systems with inputs

In the following, symmetry transformations are defined by the ability of computing new solutions of (1) using already computed ones. First, let $\Gamma$ be a group of smooth maps acting on $S$.

**Definition 4 (Definition 2 in [27]).** *We say that $\gamma \in \Gamma$ is a symmetry of (1) if for any solution $\xi(\mathbf{x}_0, p, \cdot)$, $\gamma(\xi(\mathbf{x}_0, p, \cdot))$ is also a solution.*

Using $\gamma$-symmetry, we can get a new trajectory without simulating the system but instead by just transforming the entire old trajectory using $\gamma$.

In the following definition we characterize the conditions under which a transformation is a symmetry of (1).

**Definition 5.** *The dynamic function $f : S \times P \to S$ is said to be $\Gamma$-equivariant if for any $\gamma \in \Gamma$, there exists $\rho : P \to P$ such that for all $\mathbf{x} \in S$, $\frac{\partial \gamma}{\partial \mathbf{x}} f(\mathbf{x}, p) = f(\gamma(\mathbf{x}), \rho(p))$.*

The following theorem shows that it is enough to check the condition in Definition 5 to prove that a transformation is a symmetry of (1).

**Theorem 1 (part of Theorem 10 in [27]).** *If $f$ is $\Gamma$-equivariant, then all maps in $\Gamma$ are symmetries of (1). Moreover, for any solution $\xi(\mathbf{x}_0, p, \cdot)$ and $\gamma \in \Gamma$, $\gamma(\xi(\mathbf{x}_0, p, \cdot)) = \xi(\gamma(\mathbf{x}_0), \rho(p), \cdot)$, where $\rho$ is the transformation associated with $\gamma$ in Definition 5.*

*Proof.* Let $\mathbf{y} = \gamma(\mathbf{x})$, then $\dot{\mathbf{y}} = \frac{\partial \gamma}{\partial \mathbf{x}}(\dot{\mathbf{x}}) = \frac{\partial \gamma}{\partial \mathbf{x}}(f(\mathbf{x}, p)) = f(\gamma(\mathbf{x}), \rho(p)) = f(\mathbf{y}, \rho(p))$. The second equality is a result of the derivative chain rule. The $3^{rd}$ equality uses Definition 5.

*Remark 1.* If $\gamma$ in Theorem 1 is linear, the condition in Definition 5 for a map $\gamma$ to be a symmetry becomes $\gamma(f(\mathbf{x}, p)) = f(\gamma(\mathbf{x}), \rho(p))$.

*Example 2 (Fixed-wing aircraft coordinate transformation symmetry).* Consider the fixed-wing aircraft model of Example 1. Fix $goal \in \mathbb{R}^2$ and $\theta \in \mathbb{R}$. Let $\gamma : \mathbb{R}^4 \to \mathbb{R}^4$ and $\rho : \mathbb{R}^4 \to \mathbb{R}^4$ be defined as:

$$\begin{aligned} \gamma(\mathbf{x}) = [&\mathbf{x}[0], \mathbf{x}[1] + \theta, (\mathbf{x}[2] - goal[0])\cos(\theta) + (\mathbf{x}[3] - goal[1])\sin(\theta), \\ &- (\mathbf{x}[2] - goal[0])\sin(\theta) + (\mathbf{x}[3] - goal[1])\cos(\theta)] \text{ and} \end{aligned} \tag{3}$$

$$\begin{aligned} \rho(p) = [&0, 0, (p[2] - goal[0])\cos(\theta) + (p[3] - goal[1])\sin(\theta), \\ &- (p[2] - goal[0])\sin(\theta) + (p[3] - goal[1])\cos(\theta)]. \end{aligned} \tag{4}$$

Then, for all $\mathbf{x} \in S$ and $p \in P$, $\gamma(f(\mathbf{x}, p)) = f(\gamma(\mathbf{x}), \rho(p))$, where $f$ is as in Section 2.1. The transformation $\gamma$ would change the origin of $S$ from $[0,0,0,0]$ to $[0,0,goal[0],goal[1]]$.

Then, it would rotate the third and four axes counter-clockwise by $\theta$. Moreover, $\rho$ would set the first two coordinates of the parameters to zero as they do not affect the dynamics, translate the origin of the parameter space $P$ to $[0,0,goal[0],goal[1]]$, and rotate the third and fourth axes counter-clockwise by $\theta$. For the aircraft, this means translating and rotating the plane where the aircraft and the waypoint positions reside.

#### 3.2 Symmetry and reachtubes

Computing reachtubes is computationally expensive as it requires non-trivial optimization problems and integrating non-linear functions [13,15,16,8,6]. Compared with that, transforming reachtubes is much cheaper, especially if the transformation is linear.

In our previous work [29], we showed how to get reachtubes of autonomous systems from previously computed ones using symmetry transformations. In this paper, we show how to do that for systems with parameters. This allows different modes of a hybrid system and different agents with similar dynamics to share reachtube computations. That was not possible when the theory was limited to non-parameterized systems.

**Theorem 2.** *Let (1) be $\Gamma$-equivariant. Then for any $\gamma \in \Gamma$ and its corresponding $\rho$, any $K, p, [ftime, etime]$ and $\{(X_i, [\tau_{i-1}, \tau_i])\}_{i=1}^{j}$ as a $(K, p, [ftime, etime])$-reachtube,*

$$\forall i \in [j], \mathtt{Reach}(\gamma(K), \rho(p), [\tau_{i-1}, \tau_i]) = \gamma(\mathtt{Reach}(K, p, [\tau_{i-1}, \tau_i])) \subseteq \gamma(X_i).$$

*Proof.* (Sketch) The first part $\mathtt{Reach}(\gamma(K), \rho(p), [\tau_{i-1}, \tau_i]) = \gamma(\mathtt{Reach}(K, p, [\tau_{i-1}, \tau_i]))$ follows directly from Theorem 1. The second part $\gamma(\mathtt{Reach}(K, p, [\tau_{i-1}, \tau_i])) \subseteq \gamma(X_i)$ follows from the reachtube $\mathtt{ReachTb}(K, p, [t_b, t_e])$ being an over-approximation of the exact reachset during the small time intervals $[\tau_{i-1}, \tau_i]$.

Theorem 2 says that we can transform a computed reachtube $\mathtt{ReachTb}(K, p, [t_1, t_2]) = \{(X_i, [\tau_{i-1}, \tau_i])\}_{i=1}^{j}$ to get another reachtube $\{(\gamma(X_i), [\tau_{i-1}, \tau_i])\}_{i=1}^{j}$, which is an over-approximation of the reachsets starting from $\gamma(K)$.

The results of this section subsume the results about transforming reachtubes of autonomous systems-dynamical systems without parameters as presented in [29].

### 4 Virtual system

The challenge in safety verification of multi-agent systems is that the dimensionality of the problem grows rapidly with the number of agents. However, often agents share the same dynamics. For instance, several fixed-wing aircrafts of the type described in Example 1 share the same dynamics but may have different initial conditions and follow different waypoints. This commonality has been exploited in developing specialized proof techniques [23]. For reachability analysis, using symmetry transforms of the previous section, reachtubes of one agent in one mode can be used to get the reachtubes of other modes and even other agents.

Fix a particular value $p_v \in P$ and call it the *virtual* parameter. Assume that for all $p \in P$, there exists a pair of transformations $(\gamma_p, \rho_p)$ such that $\rho_p(p) = p_v$, $\gamma_p$ is invertible, and $\gamma_p(f(\mathbf{x},p)) = f(\gamma_p(\mathbf{x}),\rho_p(p_v)) = f(\gamma_p(\mathbf{x}),p_v)$. Consider the resulting ODE:

$$\frac{d\xi}{dt}(\mathbf{y},p_v,t) = f(\xi(\mathbf{y},p_v,t),p_v). \tag{5}$$

Following [27], we call (5) a *virtual system*. Correspondingly, we call (1), the *real system* for the rest of the paper. The virtual system unifies the behavior of all modes of the real system in one representative mode, the virtual one $p_v$.

*Example 3 (Fixed-wing aircraft virtual system).* Consider the fixed-wing aircraft agent described in Example 1 and the corresponding transformations described in Example 2. Fix $p \in P$, we set *goal* in the transformation of Example 2 to $[p[2],p[3]]$ and $\theta$ to $\arctan_2(p[0]-p[2],p[3]-p[1])$ and let $\gamma_p$ and $\rho_p$ be the resulting transformations. Then, for all $p \in P$, $\rho_p(p) = [0,0,0,0]$. Hence, $p_v = [0,0,0,0]$ and the virtual system is that of Example 1 with the parameter $p = p_v$. For the aircraft, $\gamma_p$ would translate the origin of the plane to the destination waypoint and rotate its axes so that the $y$-axis is aligned with the segment between the source and destination waypoints. Hence, in the constructed virtual system, the destination waypoint is the origin of the plane. The source waypoint is the origin as well as it does not affect the dynamics.

The solutions of the virtual system can be transformed to get solutions of all other modes in $P$ using $\{\gamma_p^{-1}\}_{p\in P}$. This is shown in the following theorem.

**Theorem 3.** *Given any initial state $\mathbf{y}_0 \in S$, and any mode $p \in P$, $\gamma_p^{-1}(\xi(\mathbf{y}_0,p_v,\cdot))$ is a solution of the real system (1) with mode p starting from $\gamma_p^{-1}(\mathbf{y}_0)$. Similarly, given any $\mathbf{x}_0 \in S$, $\gamma_p(\xi(\mathbf{x}_0,p,\cdot))$ is the solution of the virtual system (5) starting from $\gamma_p(x_0)$.*

*Proof.* Lets start with the first part of the theorem. Fix $p \in P$ and let $\mathbf{x}_0 = \gamma_p^{-1}(\mathbf{y}_0)$. Using Theorem 1, $\gamma_p(\xi(\mathbf{x}_0,p,\cdot)) = \xi(\gamma_p(\mathbf{x}_0),\rho_p(p),\cdot))$ and is the solution of the real system (1). Furthermore, $\rho_p(p) = p_v$, by definition, and $\gamma_p(\mathbf{x}_0) = \gamma_p(\gamma_p^{-1}(\mathbf{y}_0)) = \mathbf{y}_0$. Hence, $\gamma_p(\xi(\mathbf{x}_0,p,\cdot)) = \xi(\mathbf{y}_0,p_v,\cdot)$. Applying $\gamma_p^{-1}$ on both sides implies the first part of the theorem. The second part is a direct application of Theorem 1.

The following corollary extends the result of Theorem 3 to reachtubes. It follows from Theorem 2.

**Corollary 1.** *Given a $K_v \subseteq S$ and a mode $p \in P$, $\gamma_p^{-1}(\texttt{ReachTb}(K_v,p_v,[t_b,t_e]))$ is a reachtube of the real system (1) with mode p starting from $\gamma_p^{-1}(K_v)$. Similarly, given any initial set $K \subset S$, $\gamma_p(\texttt{ReachTb}(K,p,[t_b,t_e]))$ is a reachtube of the virtual system (5) starting from $\gamma_p(K)$.*

Consequently, we get a solution or a reachtube for each mode $p \in P$ of the real system by simply transforming a single solution or a single reachtube of the virtual system using the transformations $\{\gamma_p\}_{p\in P}$ and their inverses. This will be the essential idea behind the savings in computation time of the new symmetry-based reachtube computation algorithm and symmetry-based safety verification algorithms presented next. It will be also the essential idea behind proving safety in the case of unbounded time and infinite number of modes.

*Example 4 (Fixed-wing aircraft infinite number of reachtubes resulting from transforming a single one).* Consider the real system in Example 1 and the virtual one in Example 3. Fix the initial set, which is represented as a hyper-rectangle, $K_r = [[1, \frac{\pi}{4}, 3, 1], [2, \frac{\pi}{3}, 4, 2]]$, the real mode $p_r = [2.5, 0.5, 13.3, 5]$, and the time bound 20 seconds. Then, similar to Example 3, we fix $\theta = \arctan_2(2.5 - 13.3, 5 - 0.5) = -1.176$ rad and $goal = [13.3, 5]$. We call the resulting transformations from Example 3, $\gamma_{p_r}$ and $\rho_{p_r}$. Let $K_v = \gamma_{p_r}(K_r)$ and $p_v = \rho_{p_r}(p_r) = [0, 0, 0, 0]$. Assume that we have the reachtube $rtube_r = \texttt{ReachTb}(K_r, p_r, T)$. Then, using Corollary 1, we can get $rtube_v = \texttt{ReachTb}(K_v, p_v, T)$ by transforming $rtube_r$ using $\gamma_{p_r}$. The benefit of the corollary appears in the following: for any $p \in P = \mathbb{R}^4$, we can get the corresponding reachtube $\texttt{ReachTb}(\gamma_p^{-1}(K_v), p, T)$ by transforming $rtube_v$ using $\gamma_p^{-1}$.

The projection of $K_v$ on its last two coordinates $K_v[2:3]$ represents the possible initial position of the aircraft in the plane relative to the destination waypoint. It would be a rotated square with angle $\theta$. The distance from $K_v[2:3]$ center to the origin would be equal to the distance from $K[2:3]$ center to the destination waypoint. Moreover, the angle between the $y$-axis and the line connecting the origin with the center of $K_v[2:3]$ would be equal to the angle from the segment connecting the source and destination waypoints to the line connecting the destination waypoint with the center of $K[2:3]$. On the other hand, $K_v[0] = K[0]$ and $K_v[1] = K[1] + \theta$.

In summary, the absolute positions of the aircraft and waypoints do not matter. What matters is their relative positions. The virtual system stores what matters and whenever a reachtube is needed for a new absolute position, we can transform it from the virtual one.

### 5 Symmetry-based verification algorithm

In this section, we introduce a novel safety verification algorithm, `symSafetyVerif`, which uses existing reachability subroutines, but exploits symmetry, unlike existing algorithms. In our earlier work [29], we introduced reachtube transformations using symmetry for single mode dynamical systems. Here, we extend the method across modes, introduce the virtual system, and develop the corresponding verification algorithm.

In Section 5.1, we define *tubecache*—a data-structure for storing reachtubes; in 5.2, we present the symmetry-based reachtube computation algorithm `symComputeReachtube` that reuses reachtubes stored in *tubecache*; finally, in 5.3, we define the *safetycache* data-structure which stores previously computed safety verification results. These results would be used by the `symSafetyVerif` algorithm.

#### 5.1 *tubecache*: shared memory for reachtubes

We show how we use the virtual system (5) to create a shared memory for the different modes of the real system (1) to reuse each others' computed reachtubes. We call this shared memory *tubecache*.

**Definition 6.** *A tubecache is a data structure that stores a set of reachtubes of the virtual system (5). It has two methods: getTube, for retrieving stored tubes and storeTube, for storing a newly computed one.*

The function getTube returns a set of reachtubes $\{\texttt{ReachTb}(K_i, p_v, [0, T_i])\}_{i \in [h]}$, for some $h \in \mathbb{N}$, that are already stored in *tubecache*. Moreover, the union of $K_i$s is the largest subset of $K$ that can be covered by the initial sets of the reachtubes in *tubecache*. Formally,

$$tubecache.\texttt{getTube}(K) = \underset{\{\texttt{ReachTb}(K_i, p_v, [0, T_i]) \in tubecache\}_i}{\text{argmax}} \text{Vol}(K \cap \cup_i K_i), \tag{6}$$

where $\text{Vol}(\cdot)$ is the Lebesgue measure of the set. Note that for any $K \subset \mathbb{R}^n$, a maximizer of (6) would be the set of all reachtubes in *tubecache*. However, this is very inefficient and it would be too conservative to be useful for checking safety. Therefore, getTube should return the minimum number of reachtubes that maximize (6). Note that the reachtubes in *tubecache* may have different time bounds. We will truncate or extend them when used.

#### 5.2 symComputeReachtube: symmetry-based reachtube computation

Given an initial set $K \subset S$, a mode $p \in P$, and time bound $T$, there are dozens of tools that can return a $\texttt{ReachTb}(K, p, [0, T])$. See [13,8,9] for examples of such tools and [26] for a comprehensive survey. We denote this procedure by $\texttt{computeReachtube}(K, p, [0, T])$.

Whenever a reachtube is needed, instead of calling computeReachtube, we will use symmetry to retrieve corresponding reachtubes that are already stored in *tubecache* and only compute what is not stored. We introduce Algorithm 1 which implements this idea and name it symComputeReachtube.

It takes as input the initial set of the virtual system $K_v$, the time bound $T$, and *tubecache*. It returns a reachtube of the virtual system starting from $K_v$ and running for $T$ time units. Hence, to get a reachtube of the real system starting from an initial set $K$ and having a mode $p$ and time bound $T$, we transform $K$ using $\gamma_p$ to get $K_v$, call symComputeReachtube, and transform the result using $\gamma_p^{-1}$.

First, it initializes *restube*$_v$ as an empty tube of the virtual system (5) to store the result in line 2. It then gets the reachtubes from *tubecache* that corresponds to $K_v$ using the getTube method in line 3. Now that it has the relevant tubes in *storedtubes*, it adjusts their lengths based on the time bound $T$. For a retrieved tube with a time bound less than $T$ in line 5, symComputeReachtube extends the tube for the remaining time using computeReachtube in lines 6-7, store the resulting tube in *tubecache* instead of the shorter one in line 8. If the retrieved tube is longer than $T$ (line 9), it trims it in line 10. However, we keep the long one in the *tubecache* to not lose a computation we already did. Then, the tube with the adjusted length is added to the result tube *restube*$_v$ in line 11.

The union of the initial sets of the tubes retrieved *storedtubes* may not contain all of the initial set $K_v$. That uncovered part is called $K'_v$ in line 12. The reachtube starting from $K'_v$ would be computed from scratch using computeReachtube in line 13, stored in *tubecache* in line 14, and added to *restube*$_v$ in line 15. The resulting tube of the virtual system (5) is returned in line 16. This tube would be transformed by the calling algorithm using $\gamma_p^{-1}$ to get the corresponding tube of the real system (5).

**Theorem 4.** *The output of Algorithm 1 is an over-approximation of the reachtube* $\texttt{ReachTb}(K_v, p_v, [0, T])$.

**Algorithm 1** `symComputeReachtube`

**input:** $K_v, T, tubecache$
$restube_v \leftarrow \emptyset$
$storedtubes \leftarrow tubecache.\texttt{getTube}(K_v)$
**for** $i \in [|storedtubes|]$ **do**
  **if** $storedtubes[i].T < T$ **then**
    $(K_i, [\tau_i, T_i]) \leftarrow storedtubes[i].end$
    $tube_i \leftarrow storedtubes[i] \frown \texttt{computeReachtube}(K_i, p_v, [0, T - \tau_i])$
    $tubecache.\texttt{storeTube}(tube_i)$
  **else if** $storedtubes[i].T > T$ **then**
    $tube_i \leftarrow storedtubes[i].truncate(T)$
  $restube_v \leftarrow restube_v \cup tube_i$
$K_v' \leftarrow K_v \setminus \cup_i storedtubes[i].K$
$tube' = \texttt{computeReachtube}(K_v', p_v, [0, T])$
$tubecache.\texttt{storeTube}(tube')$
$restube_v \leftarrow restube_v \cup tube'$
**return:** $restube_v$

*Proof.* The function `computeReachtube` always returns over-approximations of the reachset from a given initial set and for a given time bound. The set *restube* contains reachtubes that were computed by `computeReachtube` at some point. There are three types of reachtubes in *restube*:

1. When the time bound $T_i$ of the stored reachtube $storedtubes[i]$ is less than $T$, we need to extend $storedtubes[i]$ until time $T$ by concatenating the original tube with $\texttt{computeReachtube}(K_i, p_v, [0, T - \tau_i])$, where $(K_i, [\tau_i, T_i])$ is the last pair in $storedtubes[i]$. The result is a valid $(storedtubes[i].K, p_v, [0, T])$-reachtube.
2. When the time bound $T_i$ of the stored reachtube $storedtubes[i]$ is more than $T$, the truncated reachtube is also a valid $(storedtubes[i].K, p_v, [0, T])$-reachtube.
3. For $K_v'$ that is not contained in the union of the initial sets in *storedtubes*, the function `computeReachtube` will return a valid $(K_v', p_v, [0, T])$-reachtube.

The union of the initial sets of the tubes in *storedtubes* and $K_v'$ contains $K_v$, so the union of the reachtubes the algorithm returns a $(K_v, p_v, [0, T])$-reachtube.

The importance of `symComputeReachtube` lies in that if a mode $p$ required a computation of a reachtube and the result is saved in *tubecache*, another mode with a similar scenario with respect to the virtual system would reuse that tube instead of computing one from scratch. Moreover, reachtubes of the same mode might be reused as well if the scenario was repeated again.

#### 5.3 Bounded time safety

In this section, we show how to use *tubecache* and `symComputeReachtube` of the previous section for bounded and unbounded time safety verification of the real system (1). We consider a scenario where the safety verification of multiple modes of the real system (1)

starting from different initial sets and for different time horizons is needed. We will use the virtual system (5) and the transformations $\{\gamma_p\}_{p \in P}$ to share safety computations across modes, initial sets, time horizons, and unsafe sets.

We first introduce *safetycache*, a shared memory to store the results of intersecting reachtubes of the virtual system (5) with different unsafe sets. It will prevent repeating safety checking computations of different modes under similar scenarios and can be used in finding unbounded time safety properties of the real system (1).

**Definition 7.** *A safetycache is a data structure that stores the results of intersecting reachtubes of the virtual system (5) with unsafe sets. It has two functions:* `getIntersect`*, for retrieving stored results and* `storeIntersect`*, for storing a newly computed one.*

Given an initial set $K_v$, a time bound $T$, and an unsafe set $U_v$, the reachtube *rtube* $= \texttt{ReachTb}(K_v, p_v, [0,T])$ is unsafe if there is another one *rtube*$' = \texttt{ReachTb}(K_v', p_v, [0,T'])$, is unsafe, and is an under-approximation of *rtube*. Similarly, if *rtube*$'$ is an over-approximation of *rtube* and is safe, then *rtube* is safe. Formally, the `getIntersect` function of *safetycache* returns the truth value of the predicate $\texttt{ReachTb}(K_v, p_v, [0,T]) \cap U_v = \emptyset$ if a subsuming computation is stored, and returns $\perp$, otherwise.

Formally, *safetycache*.$\texttt{getIntersect}(K_v, T, U_v) =$

$$\begin{cases} 0, \text{ if } \exists\, K_v', T', U_v' \mid K_v \supseteq K_v', T \geq T', U_v \supseteq U_v', \mathit{safetycache}(K_v', T', U_v') = 0, \\ 1, \text{ if } \exists\, K_v', T', U_v' \mid K_v \subseteq K_v', T \leq T', U_v \subseteq U_v', \mathit{safetycache}(K_v', T', U_v') = 1, \text{ and} \\ \perp, \text{ otherwise,} \end{cases}$$

where 0 means *unsafe* and 1 means *safe*.

It is equivalent to check the intersection of a reachtube of the real system (1) with an unsafe set $U$ and to check the intersection of the corresponding reachtube and unsafe set of the virtual one. This is formalized in the following lemma.

**Lemma 1.** *Consider an unsafe set $U \subseteq \mathbb{R}^n \times \mathbb{R}^+$ and rtube* $= \texttt{ReachTb}(K, p, [t_1, t_2])$. *Then, for any invertible $\gamma : \mathbb{R}^n \to \mathbb{R}^n$, rtube $\cap\, U \neq \emptyset$ if and only if $\gamma(\mathit{rtube}) \cap \gamma(U) \neq \emptyset$.*

Now that we have established the equivalence of safety checking between the real and virtual systems, we present Algorithm 2 denoted by `symSafetyVerif`. It uses *safetycache*, *tubecache*, and `symComputeReachtube` in order to share safety verification computations across modes. The method `symSafetyVerif` would be called several times to check safety of different scenarios and *safetycache* and *tubecache* would be maintained across calls.

The function `symSafetyVerif` takes as input an initial set $K$, a mode $p$, a time bound $T$, an unsafe set $U$, the transformation $\gamma_p$, and *safetycache* and *tubecache* that resulted from previous runs of the algorithm.

It starts by transforming the initial and unsafe sets $K$ and $U$ to a virtual system initial and unsafe sets $K_v$ and $U_v$ using $\gamma_p$ in line 2. It then checks if a subsuming result of the safety check for the tuple $(K_v, T, U_v)$ exists in *safetycache* using its method `getIntersect` in line 3. If it does exist, it returns it directly in line 8. Otherwise, the approximate reachtube is computed using `symComputeReachtube` in line 5. The returned tube is intersected with $U_v$ in line 6 and the result of the intersection is stored in *safetycache* in line 7 and returned in line 8.

**Algorithm 2** `symSafetyVerif`

1: **input:** $K, p, T, U, \gamma_p, safetycache, tubecache$
2: $K_v \leftarrow \gamma_p(K), U_v \leftarrow \gamma_p(U)$
3: $result \leftarrow safetycache.\texttt{getIntersect}(K_v, T, U_v)$
4: **if** $result = \perp$ **then**
5: $\quad rtube \leftarrow$ `symComputeReachtube` $(K_v, T, tubecache)$
6: $\quad result \leftarrow (tube \cap U_v = \emptyset)$
7: $\quad safetycache.\texttt{storeIntersect}(K_v, T, U_v, result)$
8: **return:** *result*

**Theorem 5.** *If* `symSafetyVerif` *returns safe, then* $\texttt{ReachTb}(K, p, [0,T]) \cap U = \emptyset$.

*Proof.* From Theorem 4, if the result is not stored in *safetycache*, we know that *rtube* in line 5 is an over-approximation of $\texttt{ReachTb}(K_v, p_v, [0,T])$. Moreover, we know from Corollary 1 that $\texttt{ReachTb}(K, p, [0,T]) \subseteq \gamma_p^{-1}(rtube)$. But, from Lemma 1, we know that the truth value of the predicate $(rtube \cap U_v = \emptyset)$ is equal to that of $(\gamma_p^{-1}(rtube) \cap U = \emptyset)$ and hence *result* is *safe* if $\gamma_p^{-1}(rtube) \cap U = \emptyset$ and thus it is *safe* if $\texttt{ReachTb}(K, p, T) \cap U = \emptyset$. Finally, the stored values in *safetycache* are results from previous runs, and hence have the same property.

However, if `symSafetyVerif` returns *unsafe*, it might be that *rtube* in line 5 intersected the unsafe set because of an over-approximation error. There are two sources of such errors: first, the method `computeReachtube` used by `symComputeReachtube` can itself result in over-approximation errors. Actually, it will, most of the time [13,8]. But it may be exact too [3]. Second, the *tubecache*.`getTube` method which would return a list of tubes with the union of their initial sets strictly over-approximating the needed initial set. The first problem can be solved by asking the method `computeReachtube` to compute tighter reachtubes. Existing methods provide this option at the expense of worse computational complexity [13,8]. However, we can use symmetry in these tightening computations as well, as we did in [29]. We can also replace saved tubes in *tubecache* with newly computed tighter ones. The second problem can be solved by asking *tubecache*.`getTube` to return only the tubes with initial sets that are fully contained in the asked initial set. This would decrease the savings from transforming cached results, but it would reduce the false-positive error, saying *unsafe* while it is *safe*.

#### 5.4 Unbounded time safety

In this section, we show how infinite number of results of safety checks, i.e. results of intersections of reachtubes with unsafe sets, can be deduced from finite ones. The following corollary applies Lemma 1 to the transformations $\{\gamma_p\}_{p \in P}$ that map the different modes of the real system (1) to the unique virtual one (5).

**Corollary 2 (Infinite safety verification results from a single one).** *Fix* $U \subseteq \mathbb{R}^n$ *and* $rtube = \texttt{ReachTb}(K_v, p_v, [0,T])$. *If* $rtube \cap U = \emptyset$, *then* $\forall p \in P$, $\gamma_p^{-1}(rtube) \cap \gamma_p^{-1}(U) = \emptyset$.

The corollary means that from a single scenario safety check, i.e. an intersection operation between a reachtube $\texttt{ReachTb}(K, p_v, [0,T])$ and unsafe set $U$, we can deduce the safety of any mode $p \in P$ starting from $\gamma_p^{-1}(K)$ and running for $T$ time units with respect to the corresponding unsafe set $\gamma_p^{-1}(U)$. This would, for example, imply unbounded time safety of a hybrid automaton under the assumption that the unsafe sets of the modes are at the same relative position with respect to the reachtube. But, *safetycache* stores a number of results of such operations. We can infer from each one of them the safety of infinite scenarios. This is formalized in the following theorem which follows directly from Corollary 2.

**Theorem 6 (Infinite safety verification results from finite ones).** *For any mode $p \in P$, initial set $K \subseteq S$, time bound $T \geq 0$, and unsafe set $U \subset S \times \mathbb{R}^{\geq 0}$, such that $K \subseteq \gamma_p^{-1}(K')$, $U \subseteq \gamma_p^{-1}(U')$, and safetycache$(K', T, U') = 1$, system (1) is safe.*

As more results are added to *safetycache*, then we can deduce the safety of more scenarios in all modes. If at a given point of time, we are sure that no new scenarios would appear, we can deduce the safety for unbounded time and unbounded number of agents with the same dynamics having scenarios already covered.

*Example 5 (Fixed-wing aircraft infinite number of safety verification results from computing a single one).* Consider the initial set $K$, mode $p$, time bound $T$, their corresponding virtual ones $K_v$ and $p_v$, and the symmetry transformation $\gamma_{p_r}$ considered in Example 4. Let the unsafe set be $U = [[0, -\infty, 11.9, 5.1], [\infty, \infty, 12.9, 6.1]] \times \mathbb{R}^{\geq 0}$ and $U_v = \gamma_{p_r}(U)$. Assume that $rtube_v \cap U_v = \emptyset$ and the result is stored in *safetycache*. Then, for all $p \in P$, $\gamma_p^{-1}(rtube_v) \cap \gamma_p^{-1}(U_v) = \emptyset$.

For the aircraft, $U$ could represent a mountain. Crashing with the mountain at any speed, heading angle, and time is unsafe. $U_v$ represents the relative position of the mountain with respect to the segment of waypoints. Theorem 6 says that for any initial set of states $K$ of the aircraft and time bound $T$, if the relative positions of the aircraft, unsafe set, and the segment of waypoints are the same or subsumed by those of $K_v$, $U_v$, and the origin, we can infer safety irrespective of their absolute positions.

## 6 Experimental evaluation

We implemented a software safety verification tool for multi-agent hybrid systems based on `symComputeReachtube` using Python 3. We named it CacheReach. By hybrid, we mean systems that transition between different modes under different conditions. We tested it on a linear dynamical system and the aircraft model of Example 1, following sequences of waypoints, using DryVR [18] and Flow* [8] as reachability subroutines. Our code is available in a figshare repository [28] and has been tested on an Ubuntu virtual machine available in another figshare repository [21].

### 6.1 CacheReach: multi-agent safety verification tool

Our tool CacheReach takes as input a JSON file specifying a list of $N$ agents of dimension $n$. It also specifies the python file that contains the dynamics function $f$ of

Definition 1 and two symmetry-related functions: *symGamma* and *symGammaInv*. Given a $p \in P$ and a polytope[3] *poly* of dimension $n$ representing a set of states of the agent, *symGamma* returns $\gamma_p(poly)$, where $\gamma_p$ is the symmetry map to the virtual system. Similarly, *symGammaInv* would return $\gamma_p^{-1}(poly)$. The list of modes that the $i^{th}$ agent transition between sequentially and their corresponding transitions conditions, denoted by guards, are specified as well and denoted by $H_i$. The guard of the $j^{th}$ mode of the $i^{th}$ agent $H_i[j].guard$ is a hyper-rectangle in the state space which when the agent reaches, it transitions to the $(j+1)^{st}$ mode. The guard $H_i[j]$ has time bound $H_i[j].T$ on how long the agent can stay in the mode. Moreover, it specifies the initial set of states for each agent as a hyper-rectangle. Finally, it specifies the static unsafe set $U$ and the subset of dimensions $O \subseteq [n]$ that is relevant for dynamic safety checking between agents. If the reachtubes of two agents projected on $O$ intersect each other, it would model a collision between the agents. For example, $O$ would be $\{2,3\}$ for the aircraft model in Example 1 as $(\mathbf{x}[2], \mathbf{x}[3])$ represents its position.

CacheReach would return *unsafe* if the reachtubes of the agents starting from their initial sets of states and following the sequence of modes intersect a static unsafe set, or when projected to $O$, intersect each other. It would return *safe*, otherwise. Currently, CacheReach assumes that all agents share the same dynamics but do not interact. Hence, it has a single *tubecache* that is shared by all.

CacheReach computes the reachtubes of individual agents iteratively. It would compute the reachtube $mtube_i$ of the $j^{th}$ mode of the $i^{th}$ agent using `symComputeReachtube`. Then, it intersects it with the guard using the function *guardIntersect* to get the initial set $initset_i$ for the next mode. In addition to $initset_i$, *guardIntersect* computes the minimum and maximum times: $mintime_i$ and $maxtime_i$, respectively, at which $mtube_i$ intersects the guard. The value $mintime_i$ is the time at which a trajectory of the next mode may start at and $maxtime_i$ is the maximum such time. These values are used to check safety against time-annotated unsafe sets such as collision between agents.

The computed tube $mtube_i$ gets appended to $atube_i$ storing the full reachtube of the $i^{th}$ agent. The benefit of this method is that now all modes of all agents can be mapped to a single virtual system. They can resuse each others reachtubes using *tubecache* that is getting updated at every call to `symComputeReachtube`. Moreover, the static safety is done in the usual way.

The collision between agents is done by the function checkDynamicSafety. It takes two full reachtubes of two agents $atube_1$ and $atube_2$ along with two arrays $lookback_1$ and $lookback_2$. For agent $i$, the array $lookback_i$ consists of pairs of integers $(ind_j, timerange_j)$ specifying the index identifying the beginning of the $j^{th}$ mode tube in $atube_i$ and the uncertainty in the starting time of the trajectories from its initial set. checkDynamicSafety would use this information to time-align parts of $atube_1$ and $atube_2$ so that the intersection check happens only between two sets that may have been reached at the same time by the two agents.

[3] https://github.com/tulip-control/polytope

#### 6.2 Experimental results

We ran experiments using our tool CacheReach on two models: a 3-dimensional linear dynamical system example and the nonlinear aircraft model described in Example 1. The linear model is of the form $\dot{\mathbf{x}} = A(\mathbf{x} - p[3:5])$, where $A = [[-3,1,0],[0,-2,1],[0,0,-1]]$, $\mathbf{x} \in \mathbb{R}^3$, and $p \in \mathbb{R}^6$. We considered scenarios with single, two, and three agents for each model following different sequences of waypoints. The sequences of waypoints for the linear model are translations and rotations of a digital-$S$ shaped path. For the aircraft model, the paths are random crossing paths going north-east. In every scenario, all the agents have the same model. In the aircraft scenarios, the agent would switch to the next waypoint once its x, y position is within 0.5 units from the current waypoint in each dimension. The initial set of the aircraft was of size 1 in the position components, 0.1 in the speed, and 0.01 in the heading angle. We used Flow* [8] and DryVR [18] to compute reachtubes from scratch for the linear example. We only used DryVR for the aircraft model since our C++ Flow* wrapper does not handle a model having $\arctan_2$ in the dynamics. We ran all scenarios in CacheReach with and without using *tubecache*. The symmetry used for the aircraft was the one we showed in Example 3. For the linear model, the symmetry transformation $\gamma_p$ that was used to map the state to the virtual system was a coordinate transformation where the new origin is at the next waypoint $p[3:5]$ and rotating the $xy$-plane by the angle between the previous and the next waypoints $p[0:2]$ and $p[3:5]$ projected to the plane. We compared the computation time with and without symmetry and show the results in Table 1. The reachtubes for three nonlinear and three linear agents are shown in Figure 1. The different colors represent reachtubes of different agents, the black points represent the waypoints, the black segments connect consecutive waypoints, and the red rectangles represent the unsafe sets. The figures on the top represent the real reachtubes while those on the bottom represent the ones corresponding to the virtual system saved in *tubecache*.

Table 1: Results.

| tool \ agent model | | Linear(1,2,and 3 agents) | | | aircraft(1,2,and 3 agents) | | |
|---|---|---|---|---|---|---|---|
| Sym-DryVR | computed | 57 | 90 | 90 | 635.23 | 1181.38 | 1550.62 |
| | transformed | 42 | 165 | 264 | 20.76 | 286.62 | 501.38 |
| | time (min) | 0.093 | 0.163 | 0.187 | 3.42 | 8.2 | 10.59 |
| Sym-Flow* | computed | 39.8 | 61.14 | 66.15 | NA | NA | NA |
| | transformed | 19.2 | 84.85 | 143.85 | | | |
| | time (min) | 0.387 | 0.62 | 0.684 | | | |
| NoSym-DryVR | computed | 99 | 255 | 354 | 656 | 1468 | 2052 |
| | time (min) | 0.062 | 0.355 | 0.52 | 3.71 | 10.78 | 15.47 |
| NoSym-Flow* | computed | 59 | 151 | 210 | NA | NA | NA |
| | time (min) | 0.53 | 1.328 | 1.5 | | | |

In Table 1, we call CacheReach, when ran with DryVR while using *tubecache*, Sym-DryVR, for symmetric DryVR. We call it Sym-Flow* if we are using Flow* instead. If we are not using *tubecache*, we call them NoSym-DryVR and NoSym-Flow*, respectively. Remember in `symComputeReachtube`, some tubes may be cached but they have shorter time horizons than the needed tube. So, we compute the rest from scratch. Here, we report the fractions of tubes computed from scratch and tubes that were transformed from cached ones. Moreover, we report the execution time till the tubes are

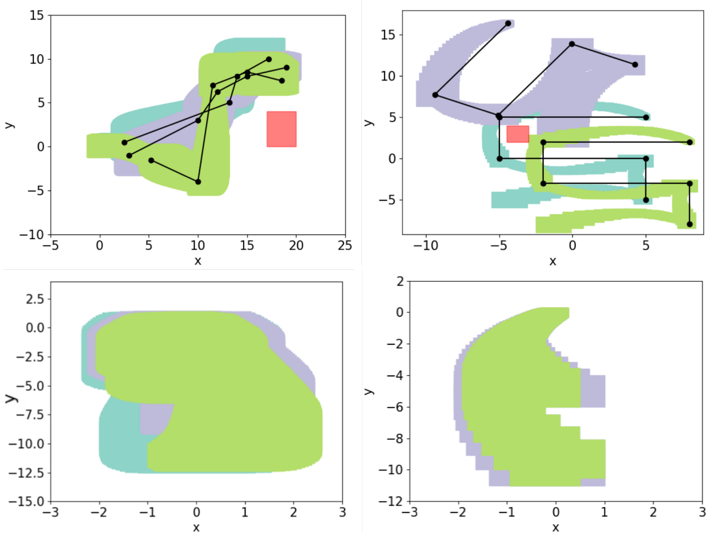

Fig. 1: Reachtubes for three fixed-wing aircrafts (left) and three linear models (right). Real reachtubes (top) vs. the virtual ones saved in *tubecache* (bottom).

computed. In the experiments, we always compute the full tubes even if it was detected to be unsafe earlier to have a fair comparison of running times. Moreover, the execution time does not include dynamic safety checking as the four versions of the experiments are doing the same computations for that purpose. We are using CacheReach in all scenarios with other reachability computation tools to decrease the degrees of freedom and show the benefits of transforming reachtubes over computing them. The Sym versions result in decrease of running time up-to 64% in the linear case with three agents. The ratio of transformed vs. computed tubes increases as the number of agents increase. This means that different agents are sharing reachtubes with each other in the virtual system. The total number of reachtubes is the same, whether *tubecache* is used or not. This means that the quality of the tubes, i.e. how tight they are, is the same whether we are transforming from *tubecache* or computing from scratch since the initial sets of modes are computed from intersections of reachtubes with guards. The fatter the reachtube is, the larger the initial set gets and the larger the number of reachtubes need to be computed.

### 7 Discussion and conclusions

In this paper, we investigated how symmetry transformations and caching can help achieve scalable, and possibly unbounded, verification of multi-agent systems. We developed a notion of *virtual system* which define symmetry transformations for a broad class of hybrid and dynamical agent models visiting waypoint sequences. Using virtual system, we present a prototype tool called CacheReach that builds a cache of reachtubes for the transformed virtual system, in a way that is agnostic of the representation of the reachsets and the reachability analysis subroutine used. Our experimental evaluation show significant improvement in computation time on simple examples and increased savings as number of agents increase.
